# Supplementary material for: Content-rich biological network constructed by mining PubMed abstracts
Source: BMC Bioinformatics. 2004 Oct 8;5:147. doi: 10.1186/1471-2105-5-147 (PMC528731; doi:10.1186/1471-2105-5-147)
Supplement: Additional File 5 — The original Chilibot query results of the term "long-term potentiation (LTP)" and 22 other terms, limiting the latest references analyzed to the years 1990, 1995, 2000, and 2004. [file 1471-2105-5-147-S5.bz2 › chilibotAdditionalFile5/ltp1995/html/TAU_ARC.html]

 


 **TAU** and **ARC** 
  
Found 4 abstracts in PubMed,  **4 abstracts were retrieved and analyzed**.  


---

 Search Google  |
 PDF files only 
|  EDU domain only 

---

**Interactive relationship** (e.g. stimulation, inhibition, etc)

**Parallel relationship** (e.g. studied together, co-existance, homology, etc.)

- Man group had significantly higher values than those in woman group in hematocrit HCT, yield stress  **tau**  0, Newtonian contribution of viscosity mu, non Newtonion contribution of viscosity eta s mu, apparent viscosity at 2.37s 1 eta s, the equilibrium value of the structural parameter A and apparent kinetic rate constant of rouleaux breakdown  **ARC** .  Ref: 2630412 Hua Xi Yi Ke Da Xue Xue Bao, 1989
- Man group had statistically higher values in HCT, yield stress  **tau**  0, Newtonian contribution of viscosity mu, non Newtonian contribution of viscosity eta s mu, apparent viscosity at 2.37 sec 1 eta s, the equilibrium value of the structural parameter A and apparent kinetic rate constant of rouleaux breakdown  **ARC**  than those in woman group.  Ref: 1932710 Biorheology, 1991
- Step movements of amplitude M min  **arc**  and pulse movements of amplitude M min  **arc**  and pulse width  **tau**  s were studied.  Ref: 4022642 Ophthalmic Physiol Opt, 1985
